# Impact of various buffers and weak bases on lysosomal and intracellular pH: Implications for infectivity of SARS‐CoV‐2

**DOI:** 10.1096/fba.2022-00062

**Published:** 2023-03-15

**Authors:** Jeffrey A. Kraut, Izaak J. Cheetham‐Wilkinson, Laura E. Swan, Massimiliano Stagi, Ira Kurtz

**Affiliations:** ^1^ Medical and Research Services VHAGLA Healthcare System, UCLA Membrane Biology Laboratory, and Division of Nephrology VHAGLA Healthcare System and David Geffen School of Medicine Los Angeles California USA; ^2^ Department of Cellular and Molecular Physiology, Institute of Systems, Molecular and Integrative Biology University of Liverpool Liverpool UK; ^3^ Division of Nephrology, Department of Medicine David Geffen School of Medicine Los Angeles California USA; ^4^ UCLA Brain Research Institute Los Angeles California USA

**Keywords:** alkalinization, carbicarb, lysosomal pH, sars‐Covid‐ 19, sodium bicarbonate, tham

## Abstract

Acidification of the cellular lysosome is an important factor in infection of mammalian cells by SARS‐CoV‐2. Therefore, raising the pH of the lysosome would theoretically be beneficial in prevention or treatment of SARS‐CoV‐2 infection. Sodium bicarbonate, carbicarb, and THAM are buffers that can be used clinically to provide base to patients. To examine whether these bases could raise lysosomal pH and therefore be a primary or adjunctive treatment of SARS‐CoV‐2 infection, we measured lysosomal and intracellular pH of mammalian cells after exposure to each of these bases. Mammalian HEK293 cells expressing RpH‐LAMP1‐3xFLAG, a ratiometric sensor of lysosomal luminal pH, were first exposed to Hepes which was then switched to sodium bicarbonate, carbicarb, or THAM and lysosomal pH measured. In bicarbonate buffer the mean lysosomal pH was 4.3 ± 0.1 (*n =* 20); *p =* NS versus Hepes (*n =* 20). The mean lysosomal pH in bicarbonate/carbonate was 4.3 ± 0.1 (*n =* 21) versus Hepes (*n =* 21), *p =* NS. In THAM buffer the mean lysosomal pH was 4.7 ± 0.07 (*n =* 20) versus Hepes (4.6 ± 0.1, *n =* 20), *p =* NS. In addition, there was no statistical difference between pH_i_ in bicarbonate, carbicarb or THAM solutions. Using the membrane permeable base NH_4_Cl (5 mM), lysosomal pH increased significantly to 5.9 ± 0.1 (*n =* 21) compared to Hepes (4.5 ± 0.07, *n =* 21); *p* < 0.0001. Similarly, exposure to 1 mM hydroxychloroquine significantly increased the lysosomal pH to (5.9 ± 0.06, *n =* 20) versus Hepes (4.3 ± 0.1, *n =* 20), *p* < 0.0001. Separately steady‐state pHi was measured in HEK293 cells bathed in various buffers. In bicarbonate pH_i_ was 7.29 ± 0.02 (*n =* 12) versus Hepes (7.45 ± 0.03, [*n =* 12]), *p* < 0.001. In cells bathed in carbicarb pH_i_ was 7.27 ± 0.02 (*n =* 5) versus Hepes (7.43 ± 0.04, [*n =* 5]), *p* < 0.01. Cells bathed in THAM had a pH_i_ of 7.25 ± 0.03 (*n =* 12) versus Hepes (7.44 ± 0.03 [*n =* 12]), *p* < 0.001. In addition, there was no statistical difference in pH_i_ in bicarbonate, carbicarb or THAM solutions. The results of these studies indicate that none of the buffers designed to provide base to patients alters lysosomal pH at the concentrations used in this study and therefore would be predicted to be of no value in the treatment of SARS‐CoV‐2 infection. If the goal is to raise lysosomal pH to decrease the infectivity of SARS‐CoV‐2, utilizing lysosomal permeable buffers at the appropriate dose that is non‐toxic appears to be a useful approach to explore.

## 1 INTRODUCTION

Infection of critical tissues, including the kidney and lungs with the virus SARS‐CoV‐2 is dependent on acidification of lysosomes.^1^, ^2^ Therefore, raising the pH of lysosomes can potentially lessen the infectivity of the virus.^3^, ^4^, ^5^ Indeed, in vitro studies suggest that alkalinization of lysosomes can potentially contribute to prevention of infection with SARS‐CoV‐2.^6^ This approach could be used alone or in combination with therapies that directly affect the virus.

In the present study, we examined the effect of exposure of a cultured mammalian cell line to various buffers designed for clinical use in the treatment of metabolic acidosis on both intracellular and lysosomal pH in order to assess the feasibility of using buffer therapy as a potential primary or adjunctive treatment for patients with SARS‐CoV‐2.

## 2 METHODS

### 2.1 Measurement of lysosomal pH

HEK293 cells expressing RpH‐LAMP1‐3xFLAG (a ratiometric sensor of lysosomal luminal pH) were utilized for measurement of lysosomal pH.^7^ Ratiometric imaging of the mCherry and pHluorin fluorophores was accomplished by excitation of the fluorophores at 561 and 488 nm, respectively and both channels acquired simultaneously to minimize misalignment between channels. Calibration curves were performed on both fixed and permeabilized cells, and on live bafilomycin A1‐treated and nigericin‐permeabilized cells by incubating cells with calibration solutions ranging from pH 4–8 as previously described.^7^ The cells were exposed to various buffers (Table 1). In all groups the cells were exposed initially to a Hepes‐buffered solution. After a steady state was reached, the cells were bathed in either bicarbonate‐buffered, carbicarb (bicarbonate/carbonate)‐buffered or THAM‐buffered solutions. In addition, the effect of the weak bases, NH_4_Cl and hydroxychloroquine (HCQ) were assessed as controls.

**TABLE 1:** Experimental solutions

	Hepes	NaHCO_3_	Carbicarb	THAM	NH_4_Cl	HQC
NaCl	140	115	115	115	135	140
CaCl_2_	1	1	1	1	1	1
MgCl_2_	1	1	1	1	1	1
K_2_HPO_4_	2.5	2.5	2.5	2.5	2.5	2.5
Dextrose	5	5	5	5	5	5
Hepes	5				5	5
NaHCO_3_		25	12.5			
Na_2_CO_3_			12.5			
THAM				25		
NH_4_Cl					5	
HQC						1
pH	7.4	7.4	7.4	7.4	7.4	7.4

*Note*: All chemical concentrations are in mM.

### 2.2 Measurement of pHi

pH_i_ of HEK293 cells was measured in cells grown on coverslips coated with PEI. The coverslips were placed in a custom designed chamber on the stage of a microscope fluorometer and loaded with the fluorescent pH_i_ probe BCECF using esterified BCECF‐AM (Life Technologies) at room temperature for 20 min. The composition of the solution used for dye loading and each of the various buffered solutions used is shown in Table 1. In each experiment, the fluorescence excitation ratio (500/440 nm; 530‐nm emission) obtained from 200 cells was averaged. The bathing solutions continuously perfused the coverslips at 2 ml/min (37°C). At the end of each experiment, the intracellular fluorescence excitation ratio was calibrated using 5 mM valinomycin (Sigma‐Aldrich) and 26 mM nigericin (Sigma‐Aldrich). In all groups as in the lysosomal measurement experiments, the cells were bathed in the Hepes‐buffered solution followed by either bicarbonate‐buffered, bicarbonate/carbonate (carbicarb)‐buffered or THAM‐buffered solutions.

## 3 RESULTS

**FIGURE 1:**
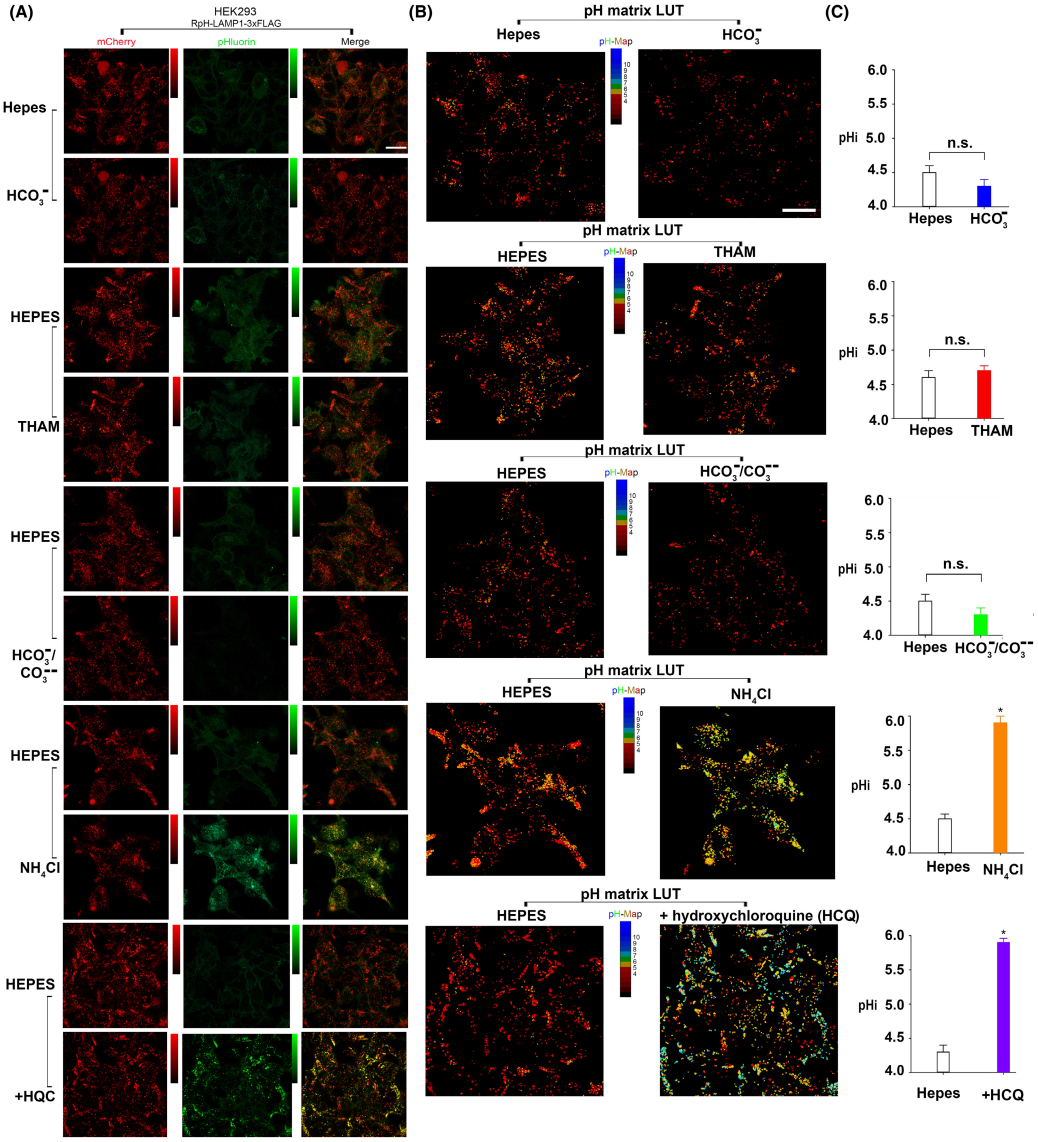
Comparison of the steady‐state lysosomal pH in Hepes, bicarbonate, carbicarb, THAM, NH_4_Cl and HCQ in HEK293 cells stably expressing RpH‐LAMP1‐3xFLAG (Scale = 20 μm). (A) Representative frames displaying cells during their initial Hepes bath and subsequent bathing with the indicated base. (B) Representative frames converted to a pH matrix, where each pixel's pH is calculated using RpH‐LAMP1‐3xFLAG before being displayed through a red (acidic) to blue (alkaline) look‐up table. (C) The steady‐state lysosomal pH in various buffers. Hepes 4.5 ± 0.1 (*n =* 20) versus HCO_3_^−^4.3 ± 0.1 (*n =* 20), *p =* NS; Hepes 4.6 ± 0.1 (*n =* 20) versus THAM 4.70 ± 0.07 (*n =* 20), *p =* NS; Hepes 4.5 ± 0.1 (*n =* 21) versus carbicarb 4.3 ± 0.1 (*n =* 21), *p =* NS; Hepes 4.50 ± 0.07 (*n =* 21) versus NH_4_Cl 5.9 ± 0.1 (*n =* 21) *p* < 0.001; Hepes 4.3 ± 0.1 (*n =* 20) versus HCQ 5.90 ± 0.06 (*n =* 20), *p* < 0.001.

Figure 1 shows the comparison of lysosomal luminal pH of HEK293 cells bathed with Hepes‐buffer versus bicarbonate, THAM, carbicarb, NH_4_Cl or HCQ containing solutions. In Hepes buffer the mean lysosomal pH was 4.5 ± 0.1 (*n* = 20) versus HCO_3_^−^ 4.3 ± 0.1 (*n* = 20), *p* = NS. The mean lysosomal pH in Hepes buffer versus THAM was 4.6 ± 0.1 (*n* = 20) and 4.70 ± 0.07 (*n* = 20) respectively, p=NS. The mean lysosomal pH in Hepes buffer versus carbicarb was 4.5 ± 0.1 (*n* = 21) and 4.3 ± 0.1 (*n* = 21) respectively, p=NS. The mean lysosomal pH in Hepes buffer versus 5 mM NH_4_Cl was 4.50 ± 0.07 (*n* = 21) versus 5.9 ± 0.1 (*n* = 21), respectively, *p* < 0.001. The mean lysosomal pH in Hepes buffer versus 1 mM HCQ was 4.3 ± 0.1 (*n* = 20) and 5.90 ± 0.06 (*n* = 20) respectively, *p* < 0.0001. Appendix S1 shows an example of the dynamic changes in lysosomal pH in response to HQC.

**FIGURE 2:**
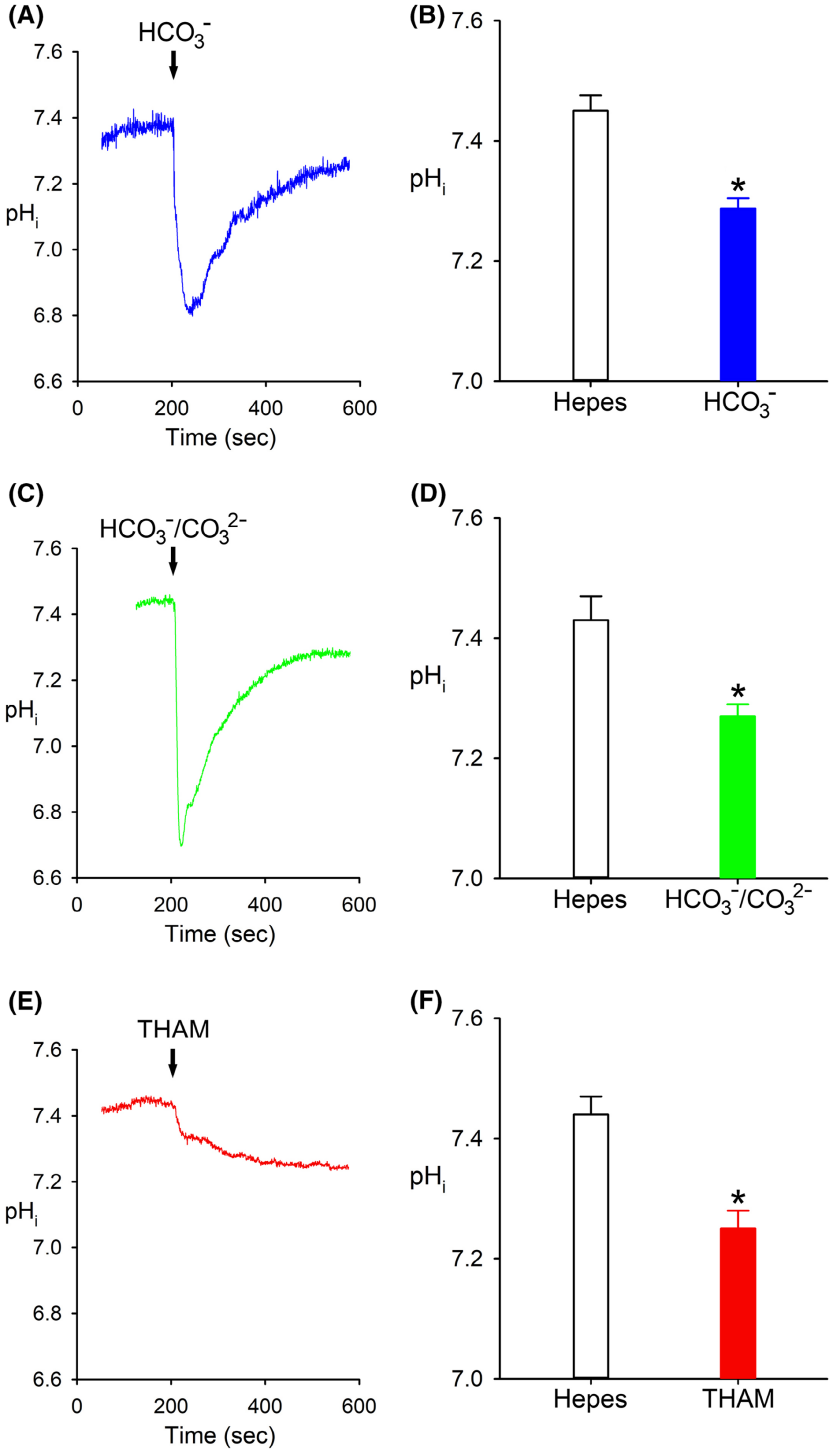
Comparison of the steady‐state pH_i_ in Hepes, bicarbonate, carbicarb and THAM. (A‐C) pHi traces showing effect of switching from Hepes buffer to HCO_3_^−^. (A) HCO_3_^−^, carbicarb (C) and THAM (E). Steady‐state pH_i_ in (B) Hepes versus HCO_3_^−^: 7.45 ± 0.03 (*n* = 12), versus 7.29 ± 0.02 (*n* = 12), *p* < 0.001 (D) Hepes versus carbicarb: 7.43 ± 0.04 (*n* = 5) versus 7.27 ± 0.02 (*n* = 5), *p* < 0.01; and (F) Hepes versus THAM: 7.44 ± 0.03 (*n* = 12) versus 7.25 ± 0.03 (*n* = 12), *p* < 0.001. In addition, there was no statistical difference in pH_i_ in bicarbonate, carbicarb or THAM solutions.

Figure 2 shows a comparison of the steady‐state pH_i_ in Hepes, bicarbonate, carbicarb and THAM‐buffered solutions. The steady‐state pH_i_ in cells bathed in bicarbonate was 7.29 ± 0.02 (*n* = 12) versus Hepes (7.45 ± 0.03, [*n* = 12]), *p* < 0.001. In cells bathed in carbicarb, pH_i_ was 7.27 ± 0.02 (*n* = 5) versus Hepes (7.43 ± 0.04, (*n* = 5)), *p* < 0.01. Cells bathed in THAM had a pH_i_ of 7.25 ± 0.03 (*n* = 12) versus Hepes (7.44 ± 0.03 [*n* = 12]), *p* < 0.001. In addition, there was no statistical difference in the pH_i_ of cells exposed to bicarbonate, carbicarb or THAM buffers.

## 4 DISCUSSION

Sodium bicarbonate, carbicarb, and THAM are buffers that have been used to provide base when it is indicated clinically. Sodium bicarbonate is most commonly used for this purpose.^8^ Although extracellular pH can be corrected with administration of sodium bicarbonate, the effect of sodium bicarbonate on lysosomal pH is unclear. Carbicarb has been used experimentally to correct both extracellular and intracellular pH, however this buffer is not yet approved for clinical use.^9^ THAM corrects extracellular pH, and in some studies also affects intracellular pH, however, similar to sodium bicarbonate, its effect on lysosomal pH is unclear.^10^ Determining whether each of these buffers can alkalinize lysosomes is necessary to determine their potential utility in treating acid‐base disorders occurring in the context of various viral infections.

In the present study, we, therefore, have addressed for the first time the effect on lysosomal pH of mammalian cells of exposure to each of these buffers. Exposure of mammalian cells to each of the aforementioned buffers had no effect on lysosomal pH. By contrast, exposure of mammalian cells to two weak bases, NH_4_Cl and HCQ^11^, ^12^ significantly increased lysosomal pH. The pH of the cytoplasm also has an independent effect on lysosome biology. At normal intracellular pH (pH_i_) lysosomes tend to localize to the center of cells, whereas changes in pH_i_ cause their redistribution. When pH_i_ is reduced below 6.5 lysosomes migrate outward and congregate at the cellular edges. In contrast, alkalinization of the cytosol causes the lysosomes to again congregate at the cellular center.^13^ In the present study, the distribution of lysosomes is likely to have remained unchanged given that steady‐state pH_i_ was not significantly different when using bicarbonate, carbicarb or THAM buffer.

The impact of our findings on potential novel treatments of SARS‐CoV‐2 needs to be viewed in light of the contribution of acidification of the lysosome to infection with this virus.^14^ Initially, viruses bind to various cell surface receptors and then undergo membrane fusion either directly at the plasma membrane or after endocytic uptake.^15^ HIV and influenza fuse directly at the plasma membrane,^16^ however both mechanisms of entry are feasible for CoV infections depending on the receptors and proteases expressed at the cell surface.^1^ Different CoVs interact with various receptors for entry. Although angiotensin converting enzyme 2 (ACE2) is a well‐studied receptor for SARS‐CoV‐2, other receptors and co‐receptors are involved.^17^ To gain entry, CoVs also require proteolytic processing of the viral envelope spike protein by host cell proteases.^18^ These viruses can directly fuse at the cell surface if the spike protein is cleaved by a surface protease like TMPRSS2,^19^ or utilizes an endosome‐lysosomal route for fusion, where the Spike protein is primed by cysteine protease cathepsins.^20^

Regarding the role of the acidic endosome‐lysosomal pathway in infection, cathepsins function optimally in a low pH environment.^21^ The dynamin and clathrin‐independent CLIC/GEEC (CG) endocytic pathway,^22^ is also pH‐dependent. Vacuolar ATPases (V‐ATPases), play a key role in the formation of CG endosomes.^23^ In contrast, the clathrin‐mediated endocytic pathway (CME) is unaffected by V‐ATPase inhibition.^24^ Homotypic fusion of nascent CG endosomes (called CLICs–clathrin‐independent carriers) forms acidic early endosomes in the CG pathway (GEECs–GPI anchored protein enriched early endosomal compartments) with an estimated luminal pH of 6.0.^24^ SARS‐CoV has also been reported to enter cells through a dynamin and clathrin‐independent endocytic pathway. In the context of SARS‐CoV‐2 entry and infection, the endocytosis of its receptor binding domain (RBD) is via the CG pathway in cells lacking ACE2.^25^ In cells overexpressing ACE2 the RBD utilizes both the CG and CME pathways. Endosomal acidification inhibitors (BafilomycinA1, niclosamide and NH_4_Cl) block viral membrane fusion and entry and alkalinize the endosomal pH and block RBD uptake (similar to CG cargo uptake). CQ blocks early steps of Spike‐pseudo‐virus entry but does not alter RBD uptake and the endocytic pH is minimally affected. Chloroquine (CQ) has been hypothesized to function to inhibit SARS‐CoV‐2 infections altering ACE2 glycosylation and activation, by affecting the ER‐stress response and by blocking a viral protein needed for Spike activation.

Neutralizing the lysosome pH obstructs endosomal trafficking, impedes sorting mechanisms, and inhibits the activity of the endosomal proteases.^26^ The pH within the lysosomal also modulates the rate of membrane fusion. The half‐maximal rate of fusion is found at a pH of 5.5 and the pH optimum is 5.0.^16^ Furthermore, little or no fusion occurs when lysosomal pH is increased to ≥ 6.0.^16^ Therefore, targeting lysosomal acidification and their acidic pH‐dependent proteases by administering therapeutic agents that raise the pH of lysosomal compartments would theoretically be beneficial in treating SARS‐CoV‐2 infection. As noted, evidence for this possibility has been gleaned from in vitro studies in which Vero cells infected with SARS‐CoV‐2 were exposed to the weak bases CQ, HCQ, and NH_4_Cl, displayed reduced infectivity with SARS‐CoV‐2.^27^

Given this, utilization of weak bases that permeate the lysosomal membrane might be an effective approach to treating patients with SARS‐CoV‐2. NH_4_Cl although efficacious in this regard in vitro^12^ has several disadvantages if given in vivo. First NH_4_^+^ enters the urea cycle and consumes bicarbonate in hepatocytes resulting in acidification of hepatic venous blood and a subsequent extracellular metabolic acidosis.^28^ NH_4_Cl also has a known effect on pH_i_.^29^ Initially pH_i_ increases because of the rapid entry of NH_3_. NH_4_^+^ enters more slowly causing a time dependent decrease in pH_i_. In general, mammalian cells possess various H^+^ and bicarbonate‐coupled transport systems that regulate pHi preventing sustained pHi changes.

The diprotic weak‐bases CQ (pKa1 = 8.1, pKa2 = 10.2) and HCQ (pKa1 = 8.3, pKa2 = 9.7) are antimalarial drugs that are present in protonated and unprotonated forms and unlike NH_4_^+^ do not consume bicarbonate metabolically. Unprotonated forms of CQ and HCQ are uncharged and can freely diffuse across lipid membranes into the lumen of organelles which acidify their lumens with a vacuolar H^+^‐ATPase such as the lysosome. The unprotonated forms bind luminal protons which results in an increase in lumen pH whose magnitude depends on the amount weak base that enters and ability of the lysosome to oppose the pH change by enhancing their H^+^‐ secretory rate. The driving force for accumulation of CQ and HCQ is proportional to the square of the hydrogen ion gradient, unlike a monoprotic weak base like NH_4_Cl, which is proportional to the proton gradient.^30^ Once protonated, CQ and HCQ are charged and get trapped in the lumen.^30^ The ability of CQ and HCQ to raise lysosomal pH suggests that these agents might be efficacious in treating SARS‐CoV‐2 and potentially other viral disorders that require acidic lysosomes for viral infectivity. The current literature, however, in general does not show a significant impact of HCQ on clinical infection.^31^, ^32^ Whether the pH of lysosomes in these studies was increased sufficiently and the dose dependence of the change in lysosomal pH clinically remains to be determined. It is conceivable that the doses administered thus far to treat patients with SARS‐CoV‐2 have not been sufficient to increase lysosomal pH to the necessary values to deter viral infection. This is a difficult issue to examine in vivo.

Lysosomal pH can also be altered by inhibiting the V‐type H^+^‐ATPase.^33^ Exposure to bafilomycin inhibits the V‐type H^+^‐ATPase and also raises the lysosomal pH predictably deterring viral infection.^34^ Clinical use of bafilomycin has generally been avoided because of its high toxicity profile. However, recent studies of its potential use in the treatment of pediatric lymphoblastic leukemia have revealed it is effective in killing the cancerous cells at concentrations that in in vivo mouse toxicity studies have been shown to be safe.^35^ Future studies of its use in the treatment of SARS‐CoV‐2 therefore seems to be indicated. In vitro studies have revealed that exposure of tracheal epithelial cells to the H^+^‐K^+^‐ATPase inhibitor lansoprazole raised the pH of cellular lysosomes and reduced infectivity of rhinoviruses, presumably by inhibiting the H^+^‐ATPase.^36^ Whether administration of the appropriate doses of proton pump inhibitors would be successful in vivo in treating SARS‐CoV‐2 infection remains to be determined.

We conclude since acidification of the lysosome is a critical factor in infectivity of cells by SARS‐CoV‐2, elevating lysosomal pH for a sufficient period of time above a critical level would intuitively be a fruitful approach clinically to treat patients. Buffers such as bicarbonate, carbicarb, or THAM at concentrations used to treat metabolic acidosis can perturb pH_i_ of mammalian cells in some studies, but do not appear to affect lysosomal pH. Higher concentrations of these buffers could potentially increase lysosomal pH sufficiently but could result in additional toxicity.

The structural features of other compounds that can enter the lysosome and raise its pH has been examined by several investigators.^24^, ^37^ For the most part, similar to chloroquine they are permeable weak bases including doxorubicin, mitoxantrone, or clomipramine. Examination of the impact of these compounds on patients with viral disease, either alone or with one of the buffers, could provide evidence that this approach could potentially benefit not only patients with SARS‐CoV‐2, but patients with other viral diseases if administered at both the appropriate time and dose in the course of infection.

## AUTHORS CONTRIBUTIONS

The contributions of the authors were as follows: Jeffrey A. Kraut assisted in the design of the studies and assisted in the writing of the manuscript. Ira Kurtz assisted in the design of the studies and assisted in the writing of the manuscript. Drs Cheetham‐Wilkinson, Swan, and Stagi helped perform the studies and assisted in the writing of the manuscript. All authors approved the final manuscript.

## DISCLOSURES

The authors declare that they have no conflict of interest.

## Supporting information


Appendix S1.

Appendix S2.

Appendix S3.

## Data Availability

The data that support the findings of this study are available from I Kurtz upon reasonable request.
