# Supplementary figures and images for: Impact of various buffers and weak bases on lysosomal and intracellular pH: Implications for infectivity of SARS‐CoV‐2
Source: FASEB Bioadv. 2023 Mar 15;5(4):149–55. doi: 10.1096/fba.2022-00062 (PMC10068769; doi:10.1096/fba.2022-00062)

## Alkalinization of Lysosome pH

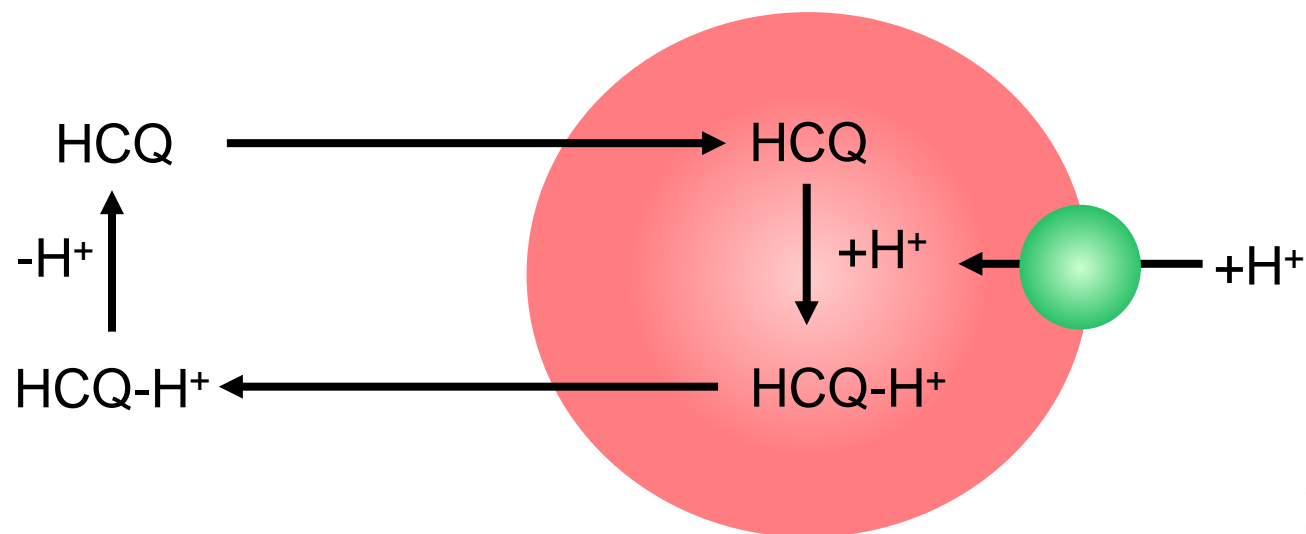

## No Change in Lysosome pH

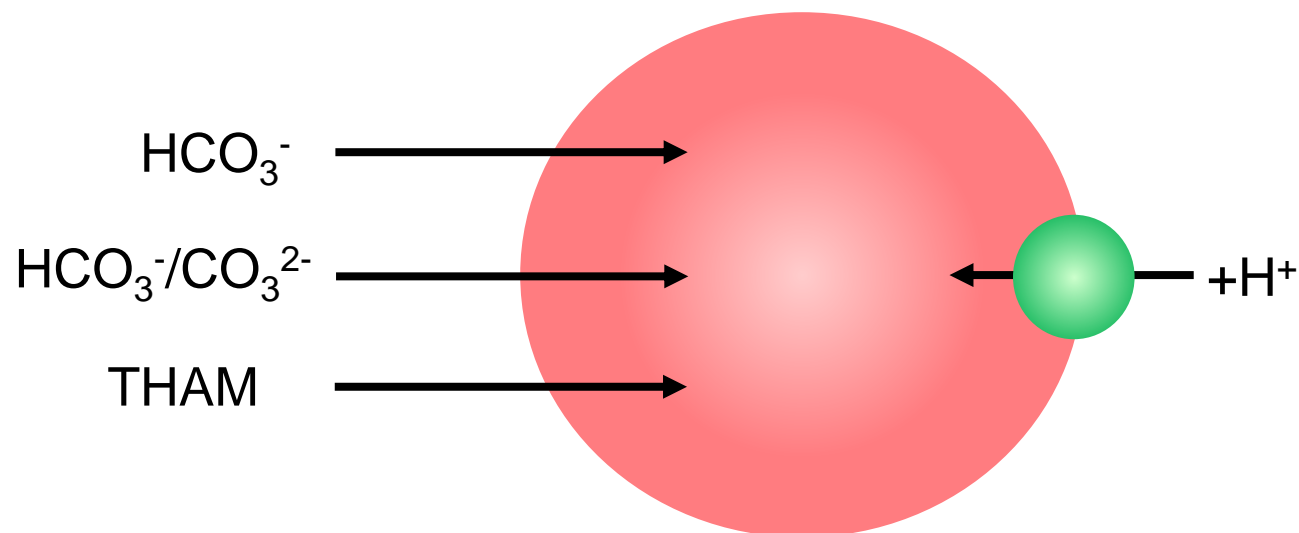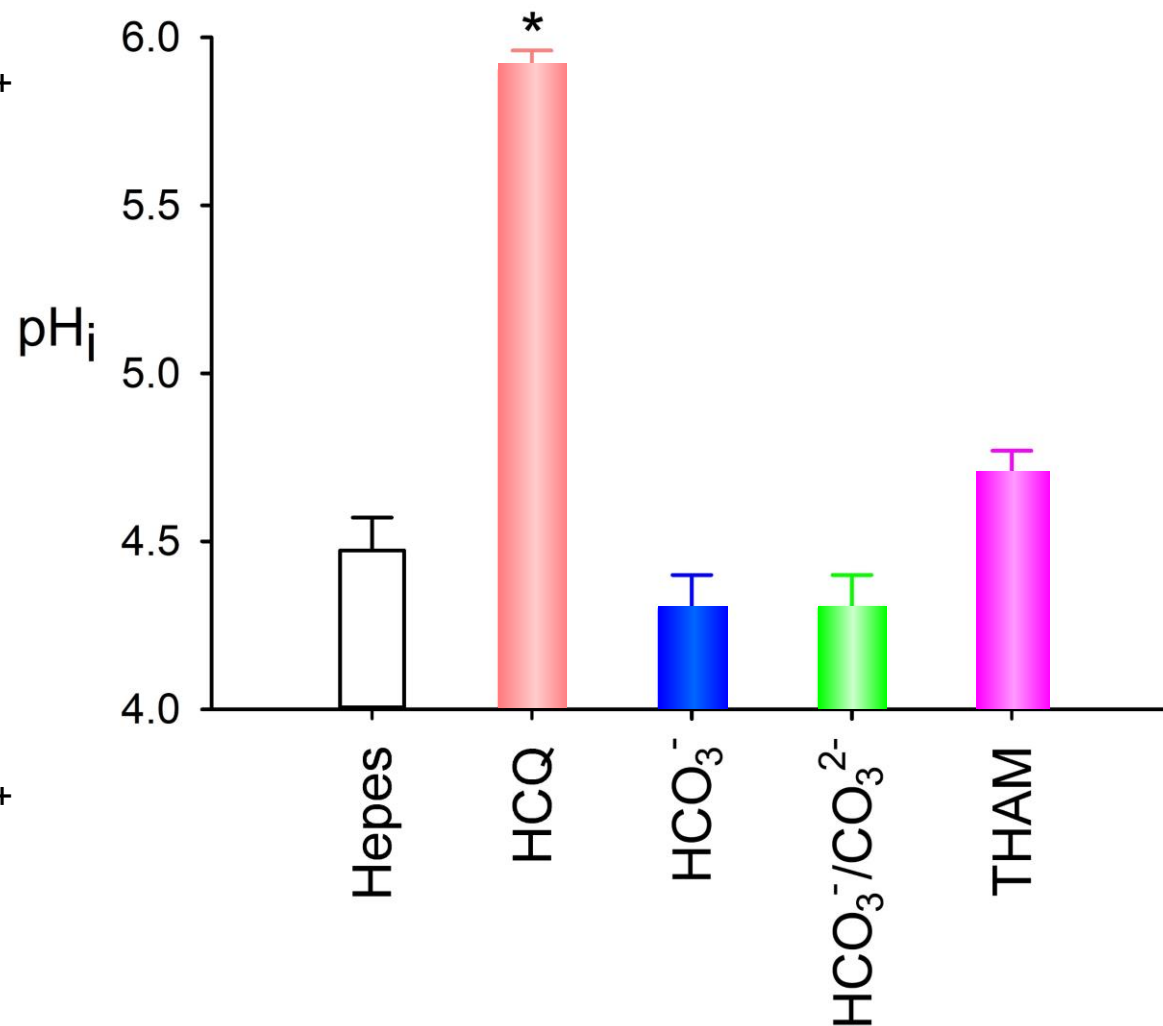

Supplement: Supplementary file 1 — Appendix S1. [file FBA2-5-149-s003.pdf]
