# Supplementary material for: Impact of various buffers and weak bases on lysosomal and intracellular pH: Implications for infectivity of SARS‐CoV‐2
Source: FASEB Bioadv. 2023 Mar 15;5(4):149–55. doi: 10.1096/fba.2022-00062 (PMC10068769; doi:10.1096/fba.2022-00062)
Supplement: Supplementary file 2 — Appendix S2. [file FBA2-5-149-s002.docx]

Graphical abstract

Acidification of the lysosome is an important factor in infection of mammalian cells by SARS-CoV-2. Therefore, raising lysosomal pH would theoretically be beneficial in prevention or treatment of SARS-CoV-2 infection. The ability of various bases traditionally used to treat acid-base disorders including bicarbonate, carbicarb, and THAM, to raise lysosomal pH was examined by exposing cultured cells to fixed concentration of these bases. Lysosomal pH was measured with RpH-LAMP1-3xFLAG, a ratiometric lysosomal pH sensor. No significant change in lysosomal pH was detected with exposure to these bases. By contrast exposure of cells to weak bases such as hydroxychloroquine (HCQ) caused a significant increase in lysosomal pH.
