# Supplementary material for: Impact of various buffers and weak bases on lysosomal and intracellular pH: Implications for infectivity of SARS‐CoV‐2
Source: FASEB Bioadv. 2023 Mar 15;5(4):149–55. doi: 10.1096/fba.2022-00062 (PMC10068769; doi:10.1096/fba.2022-00062)
Supplement: Supplementary file 3 — Appendix S3. [file FBA2-5-149-s001.docx]

Supplementary Movie 1


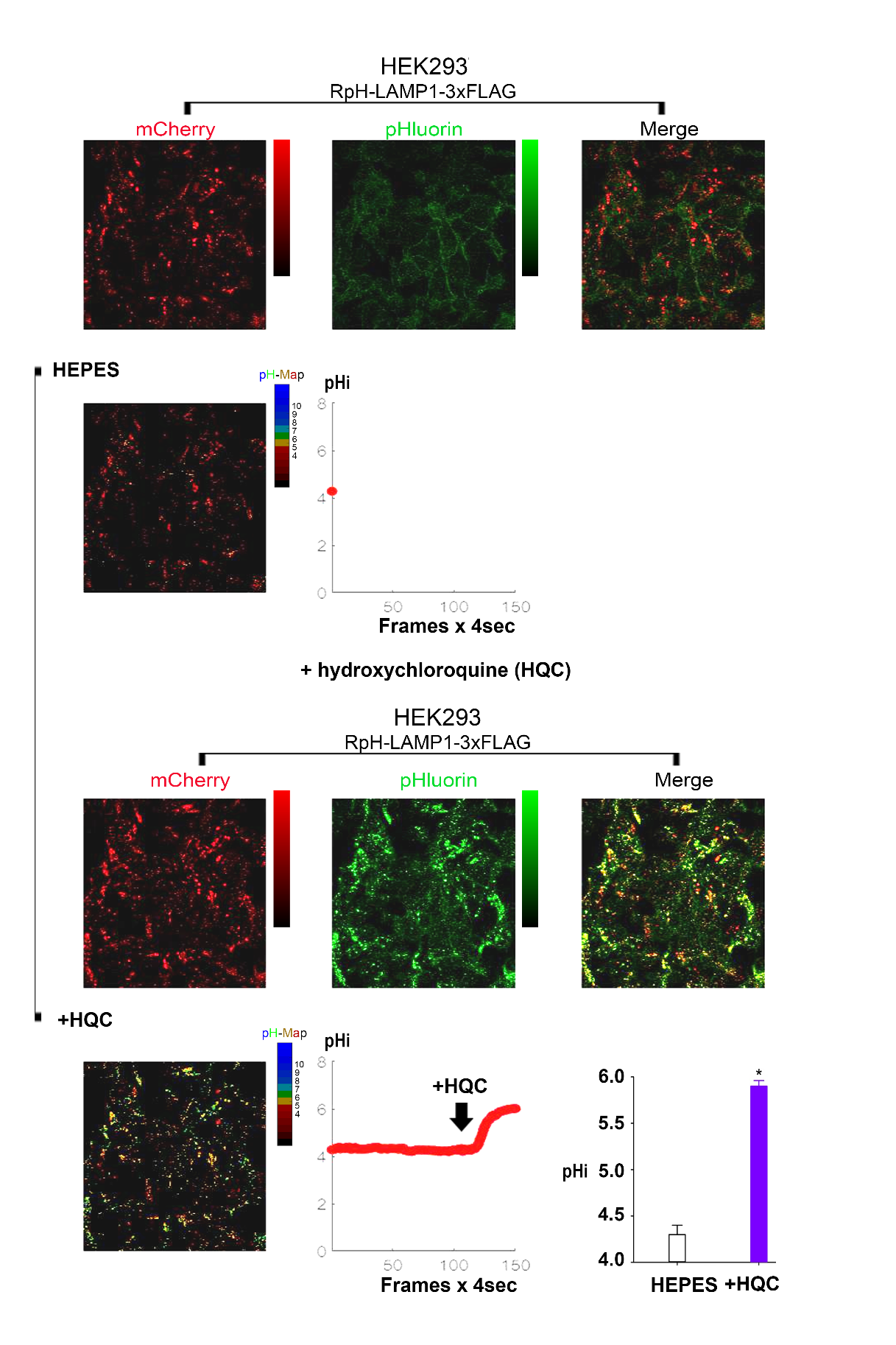


Figure legend

Supplementary Movie 1: HEK293 cells stably expressing RpH-LAMP1-3xFLAG bathed in Hepes followed by HCQ (4s per frame). Topmost frames display split (left mCherry, centre pHluorin) and merged (right) imaging channels. Bottom left shows a pH matrix, where each pixel’s pH is calculated using RpH-LAMP1-3xFLAG before being displayed through a red (acidic) to blue (alkaline) look-up table. Bottom center displays global lysosomal pH plotted over time, with indication of the point of HCQ administration. Bottom right cells bathed in HCQ had a lysosomal pH of 5.90 ± 0.06 (n=20) versus Hepes 4.3 ± 0.1 (n=20), p<0.001.
